# Prognostic value of follistatin-like 3 in human invasive breast cancer

**DOI:** 10.18632/oncotarget.15026

**Published:** 2017-02-02

**Authors:** Henrique L. Couto, Marcelo A. Buzelin, Nivaldo H. Toppa, Enrrico Bloise, Alberto J. Wainstein, Fernando M. Reis

**Affiliations:** ^1^ Division of Human Reproduction and Department of Obstetrics and Gynecology, Universidade Federal de Minas Gerais, Belo Horizonte, Minas Gerais, Brazil; ^2^ Department of Oncology, Hospital Alberto Cavalcanti, Belo Horizonte, Minas Gerais, Brazil; ^3^ Instituto Moacyr Junqueira, Belo Horizonte, Minas Gerais, Brazil; ^4^ Laboratório Analys Patologia, Belo Horizonte, Minas Gerais, Brazil; ^5^ Department of Morphology, Universidade Federal de Minas Gerais, Belo Horizonte, Minas Gerais, Brazil

**Keywords:** FSTL3, FLRG, follistatin, activin, breast cancer

## Abstract

Follistatin-like 3 (FSTL3) binds and inactivates activin, a growth factor involved with cell growth and differentiation. We have previously shown FSTL3 overexpression in invasive breast cancers, but its clinical relevance remained unexplored. Here we evaluate FSTL3 as a prognostic tool and its relation with clinical and pathological features of breast cancer. A cohort of 154 women diagnosed with invasive breast cancer between 2008 and 2012 was followed up for 5 years. Tumor samples were processed by immunohistochemistry to detect FSTL3 expression in tumor epithelium. FSTL3 expression was classified semiquantitatively and tested for possible correlation with age, menopause status, stage, tumor histological type and grade, estrogen receptor, progesterone receptor, and HER2 expression. Survival plots with Kaplan-Mayer statistics were used to assess whether FSTL3 expression predicted disease-free survival. Our findings show that FSTL3 staining was unrelated to menopausal status, histological type, disease stage, or receptor profile. However, the intensity of FSTL3 immunostaining correlated inversely with tumor size (r = -0.366, p<0.001) and with nuclear grade (p<0.01). The intensity of FSTL3 expression in the tumoral epithelium was not predictive of the disease-free survival (p = 0.991, log-rank test), even though the follow-up length and the study size were sufficient to detect a significant reduction in disease-free survival among women with stage III-IV compared to stage I-II disease (p<0.001). FSTL3 expression in invasive breast cancer is inversely associated with tumor size and nuclear grade but it does not predict disease relapse in the short term.

## INTRODUCTION

Activins are members of the transforming growth factor-β (TGF-β) superfamily that control many physiological processes such as cell proliferation and differentiation, immune responses, wound repair and various endocrine activities [1]. Like other TGF-β superfamily members, activins elicit these diverse biological responses by signaling via type I and type II receptor serine kinases [1]. More specifically, activins bind selectively to ActRIB and ActRII or ActRIIB receptor subtypes [2, 3].

These biological events are inhibited mainly by activin-binding proteins namely follistatin and follistatin-like 3 (FSTL3), the latter also known as follistatin-related protein or follistatin-related gene (FLRG) protein [1]. FSTL3 is a member of the follistatin family that differs from follistatin in lacking the third follistatin domain and a consensus heparin-binding sequence. While follistatin is predominantly found in the ovaries and pituitary, FSTL3 is widely distributed and has been found in reproductive organs as well as in the cardiovascular system, lung and skin [4]. Furthermore, FSTL3 also binds other growth factors such as myostatin, bone morphogenetic protein (BMP) 2, BMP4, BMP11 and BMP15 [1, 3, 5].

Serum activin A levels are increased in patients with metastatic cancer [6] and with anorexia/cachexia syndrome [7], and ActRIIB antagonism reverses cachexia and muscle wasting in tumor-bearing mice [8], which is evidence that activin signaling supports systemic disease progression. However, activin A inhibits growth in many types of cell and acts as a tumor suppressor in the early stages of tumorigenesis [9]. Many tumor cells and proliferative disorders escape the growth inhibitory effect of activin by acquiring mutations or loosing functionality of activin receptors [10, 11]. Thus, in most tissues activin inhibits the development of cancer and blocking of its actions may be detrimental.

Currently available data strongly suggest a role for activin-mediated signaling in morphogenesis, development, differentiation, and neoplastic transformation of the mammary gland [12–15]. Immunoassays that detect serum activin A demonstrated that median activin A levels were slightly increased in patients with breast tumors [16] and higher levels of dimeric activin A were detected in homogenates of breast cancer tissue compared to non-neoplastic tissue [16]. This is somewhat counterintuitive given that activin A inhibits and FSTL3 stimulates the *in vitro* proliferation of human breast cancer cells [13, 17, 18].

A strong nuclear expression of FSTL3 was observed in invasive breast carcinomas in contrast with the normal luminal epithelial cells in which FSTL3 was not detected [18, 19]. These observations suggest that endogenous FSTL3 contributes to tumor cell proliferation through antagonizing endogenous activin effects [18]. FSTL3 also binds and neutralizes other growth factors involved in cancer progression such as myostatin [20] and bone morphogenetic proteins [21], highlighting the need of better characterization of FSTL3 actions and prognostic value in breast cancer and other malignancies.

Despite the above evidence that FSTL3 might favor breast cancer cell proliferation and somehow be part of breast cancer development and progression, there are no studies evaluating FSTL3 in breast cancer in the clinical setting. Therefore, we designed the present study in order to evaluate FSTL3 as a prognostic tool and its relation with clinical and pathological features of breast cancer.

## RESULTS

**Figure 1:**
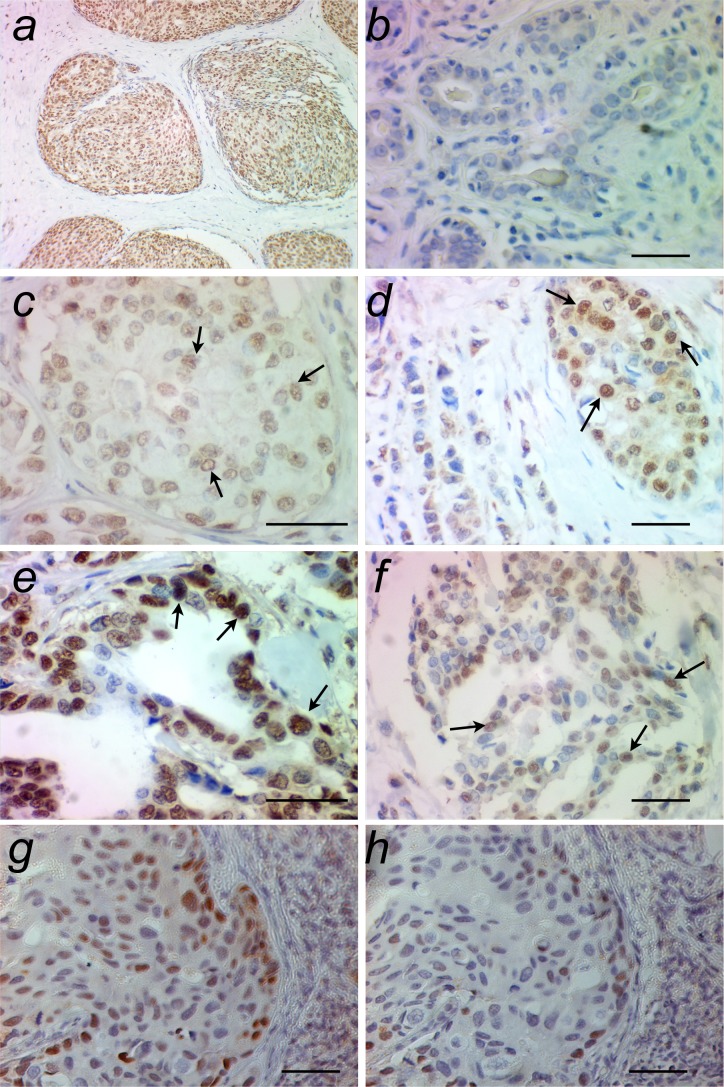
Examples of breast ductal carcinoma showing epithelial immunostaining to FSTL3 (**a**, panoramic view). The intensity of immunostaining in the tumoral epithelium (arrows) was graded as absent (**b**, score 0), mild (**c**, score 1), moderate (**d**, score 2) or intense (**e**, score 3). The percentage of tumoral cells with positive staining was graded as 0 (absent, **b**), 1 (1% to 25%), 2 (26% to 75%, **c, d**), or 3 (76% to 100%, **e**). The index was obtained by summing the intensity and the percentage scores. In the example shown in **(f)**, intensity score = 2 and percentage score = 2, then index = 2+2 = 4. The specificity of the primary antibody is demonstrated by incubation of two sequential sections of the same tumor without **(g)** and with **(h)** preadsorption of the antibody with equimolar human FSTL3 peptide. Scale bar = 50 μm.

**Figure 2:**
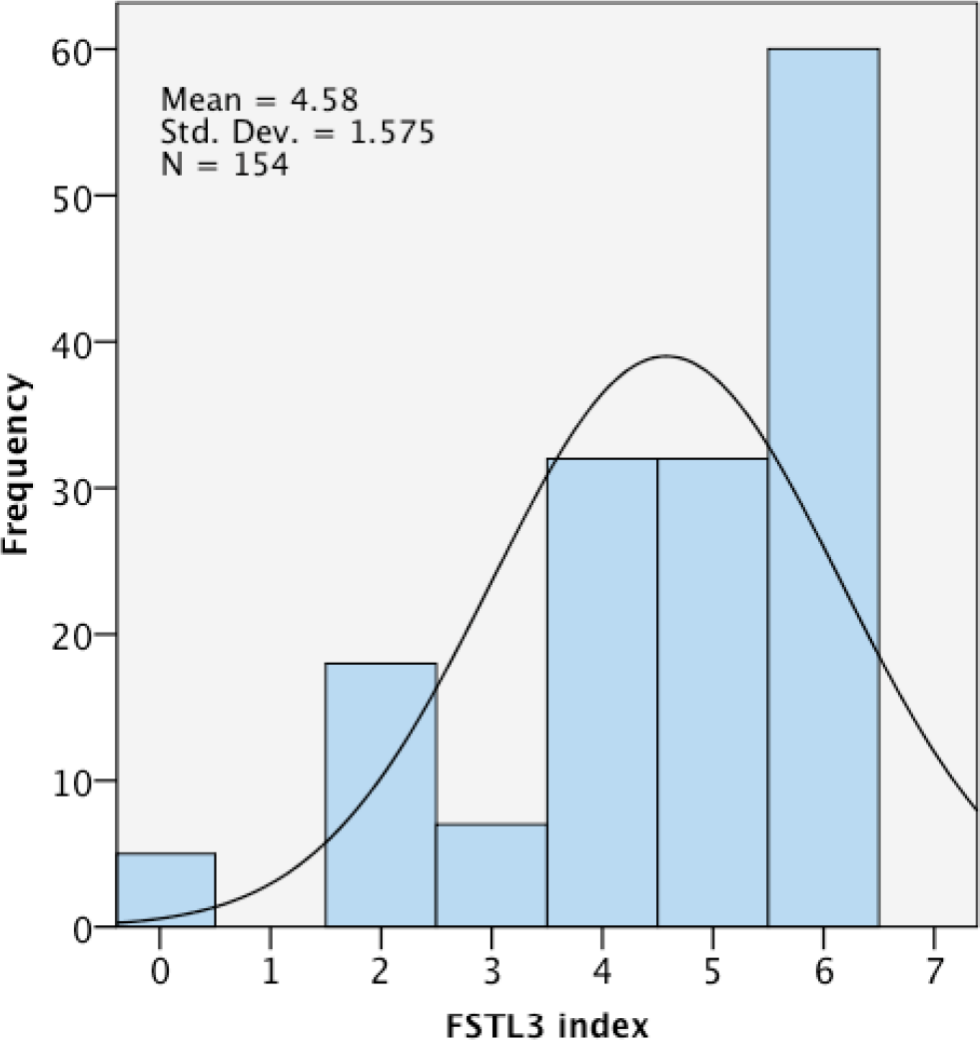
Distribution of FSTL3 immunostaining index in 154 invasive breast cancers The distribution is skewed to the right side and departs from the normal curve centered in the arithmetic mean.

All the samples were successfully evaluated by immunohistochemistry assay that localized FSTL3 staining in the nuclei of tumoral epithelial cells (Figure 1). The FSTL3 staining index ranged from 0 to 6, with a mean of 4.58 and a skewed, non-normal distribution (Figure 2).

The intensity of FSTL3 expression in the tumoral epithelium was evaluated according to clinical and histological characteristics of prognostic relevance (Tables 1 and 2). As shown in Table 1, the intensity of FSTL3 staining was unrelated to menopausal status, histological type, disease stage, or receptor profile. However, FSTL3 immunoreactivity was significantly stronger in tumors with nuclear grade 1-2 (median immunostaining index 5.0 [IQR 4.0-6.0]) than in tumors with nuclear grade 3 (median index 3.0 [2.0-4.5], p<0.001, Mann-Whitney test). The intensity of FSTL3 immunostaining also correlated inversely with tumor size (r = -0.366, p<0.001) and with the number of metastatic lymph nodes (r = -0.237, p = 0.004, Table 2). Follistatin staining did not correlate with that of FSTL3 (r = 0.06, p=0.47) although the two molecules were detectable in most specimens. Furthermore, the combined scoring of follistatin and FSTL3 did not modify the clinical correlations of FSTL3 alone.

**Table 1:** Median FSTL3 index obtained in breast cancer samples subdivided according to several patient and tumor characteristics

Variable	N	Median	Q1-Q3	P value
Menopause				
Yes	98	5.0	4.0-6.0	0.459
No	34	5.0	4.0-6.0	
Histology				
IDC NOS	130	5.0	4.0-6.0	0.801
Other types	23	5.0	4.0-6.0	
Skin invasion				
Yes	22	5.0	4.0-6.0	0.509
No	131	5.0	4.0-6.0	
Stage				
I	42	5.0	4.0-6.0	0.122
II	63	5.0	4.0-6.0	
III	44	5.0	2.5-5.5	
IV	4	5.0	4.0-6.0	
Histological grade				
1	5	5.0	4.0-6.0	0.092
2	78	5.0	4.0-6.0	
3	46	4.0	2.0-6.0	
Nuclear grade				
1-2	87	5.0	4.0-6.0	**<0. 001**
3	24	3.0	2.0-4.5	
Mitotic index				
1	7	5.0	4.5-5.5	0.300
2	93	5.0	4.0-6.0	
3	10	3.5	2.0-5.0	
ER				
Positive	116	5.0	4.0-6.0	0.625
Negative	28	5.0	4.0-6.0	
PR				
Positive	102	5.0	4.0-6.0	0.334
Negative	37	5.0	4.0-6.0	
HER2				
Positive	37	5.0	4.0-6.0	0.395
Negative	82	5.0	4.0-6.0	

P values were calculated by Mann-Whitney test.

IDC NOS: ductal carcinoma not otherwise specified

The total of subjects in each classification is less than 154 due to missing data.

**Table 2:** Spearman's correlation coefficients between FSTL3 immunostaining index and quantitative clinical variables in women with invasive breast cancer

Clinical variable	r	p
Age	0.016	0.788
Tumor size	−0.366	**<0.001**
Number of metastatic lymph nodes	−0.237	**0.004**

Table 3 summarizes the multivariate analysis of clinical and histological variables potentially affecting FSTL3 staining in tumor epithelium. The stepwise backward model started with all variables that correlated with FSTL3 at p<0.25 significance level. At the final step of the multivariate analysis, the remaining variables were nuclear grade 1-2 (adjusted odds ratio 4.97 [95% CI 1.62-15.21]) and tumor size (adjusted odds ratio 0.97 [95% CI 0.95-1.00], Table 3).

**Table 3:** Multivariate analysis of clinical and histological variables potentially affecting strong FSTL3 staining (index >4) in tumor epithelium

Variable	p	Adjusted Odds Ratio	95% CI
Nuclear grade 1-2	0.005	4.97	[1.62-15.21]
Tumor size	0.105	0.97	[0.95-1.00]
Number of metastatic lymph nodes	0.272	excluded	−
Stage	0.330	excluded	−
Histological grade	0.800	excluded	−

**Figure 3:**
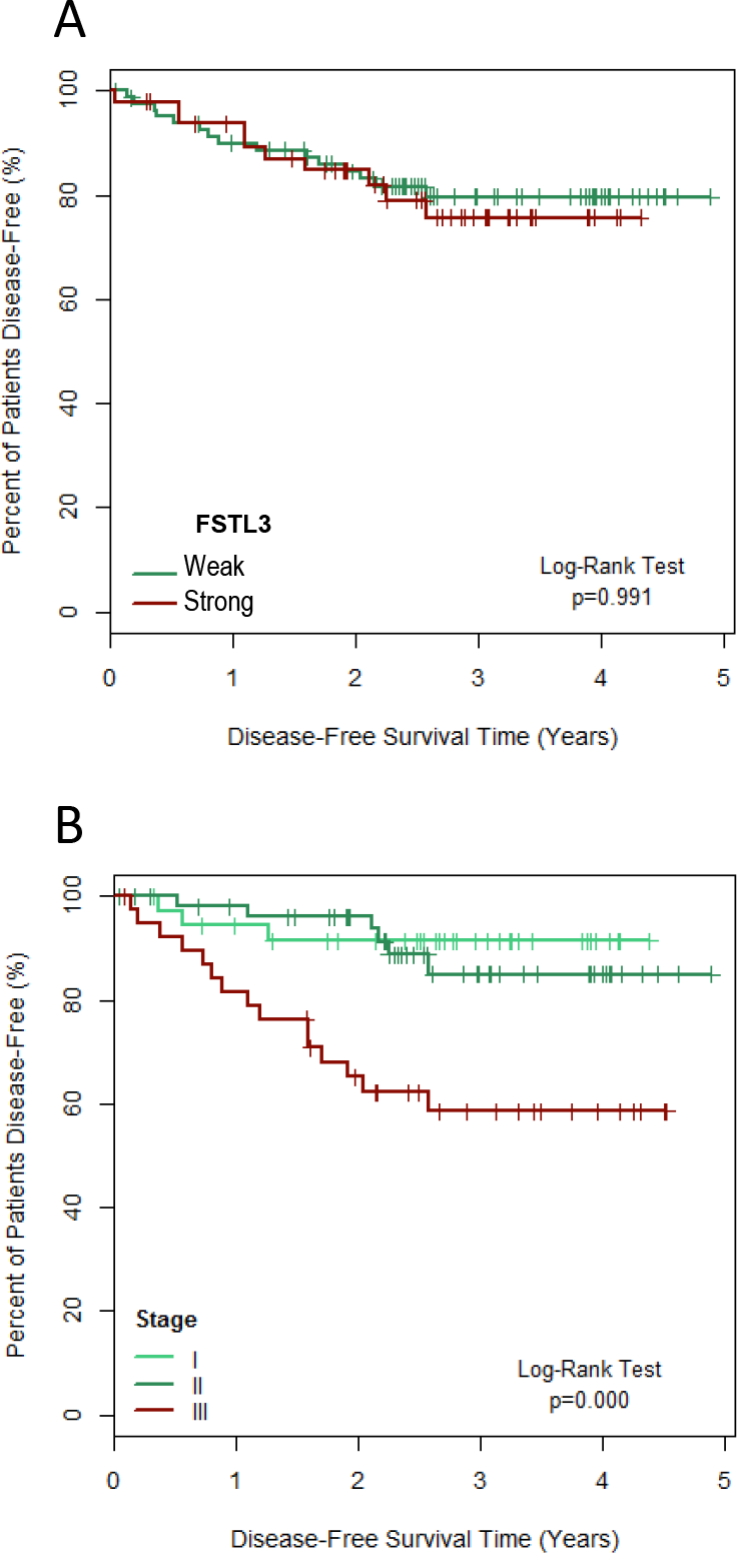
Survival plots for the disease-free time after primary surgical treatment of invasive breast cancer (n = 132) according to the tumor expression of FSTL3 **(A)** and to the disease stage **(B)**. FSTL3 expression was classified as weak (immunostaining index 0-4) or strong (index 5-6).

The 132 patients submitted to primary surgical treatment were followed during 2 to 60 months (median follow-up 31 months) and there were 25 events of local and/or systemic disease recurrence. As shown in Figure 3A, the intensity of FSTL3 expression in the tumoral epithelium was not predictive of the disease-free survival (p = 0.991, log-rank test). However, the follow-up length and the study size were sufficient to detect a significant reduction in disease-free survival among women with stage III-IV compared to stage I-II disease (p<0.001, Figure 3B).

## DISCUSSION

This study evaluated breast cancer FSTL3 expression in the clinical setting. We found that FSTL3 expression correlated to tumor nuclear grade, tumor size, and the number of reactive lymph nodes. FSTL3 expression also showed no prognostic value in breast cancer patients.

Previously, we have screened FSTL3 protein and mRNA levels in different breast proliferative disorders [19]. Invasive breast cancer specimens had higher area and intensity of FSTL3 mRNA and protein staining in tumor epithelial cells compared to other less invasive breast proliferative diseases, suggesting that FSTL3 might be involved in breast cancer progression. Here, we expand our previous findings by depicting FSTL3 expression in relation to clinical and pathological features of invasive breast cancer.

The first pathological aspect correlated to FSTL3 expression was the tumoral nuclear grade, as low and moderate nuclear grade (grade 1 or 2) tumors expressed FSTL3 with greater intensity than high nuclear grade (grade 3) tumors. Low nuclear grade tumors generally have lower growth rates [22]. Thus, our data suggest that FSTL3 expression may be more abundant in the slower proliferating tumors, challenging our previous assumption that this protein would contribute to human breast cancer growth. Since a temporal and causal relationship between FSTL3 expression and breast carcinogenesis in humans cannot be properly investigated *in vivo*, we speculate that this protein is constitutively expressed in invasive breast cancers and is down-regulated by still unknown mechanisms in high nuclear grade lesions.

The index of FSTL3 immunostaining was inversely correlated to tumor size. This is interesting since small tumors are more often detected at screening programs and they also tend to be well differentiated (lower nuclear grade) [23]. This finding is in accordance with the stronger FSTL3 expression in tumors of lower nuclear grade, which usually grow at slower rates compared to those of higher nuclear grade [22].

Although FSTL3 expression correlated inversely to nuclear grade and tumor size, it was unrelated to other known prognostic factors such as disease stage and the number of metastatic lymph nodes, the latter being excluded by the multivariate analysis. Overall, even though compelling data suggest that FSTL3 stimulates breast cancer proliferation [18], the present evidence suggests that the pattern of FSTL3 expression in the tumor epithelium does not determine the aggressiveness of the disease. Nevertheless, a biological role for this binding protein in the mechanisms of tumorigenesis is still supported by consistent experimental evidence [18] and cannot be ruled out by the lack of prognostic determinism. Actually, the present demonstration of variable FSTL3 distribution in invasive breast cancer, ranging from faint and scattered to strong and widespread, suggests that this protein might represent an endogenous mechanism of cell growth that is active in specific tumors and has the potential to be targeted therapeutically.

Despite their structural similarity, follistatin and FSTL3 are independently regulated [4]. This dissociation has been documented in breast fibroadenoma that overexpressed follistatin but not FSTL3 [19]. In the present study, FSTL3 was equally present in ER-positive and ER-negative tumors, whereas follistatin had been shown to be more abundant in ER-negative tumors [24].

Owed to its inability to bind cell surface proteoglycans, breast-synthetized FSTL3 is likely to gain the systemic circulation [3]. Similarly, the FSTL3 released by other organs has the potential to stimulate the breast in normal or in pathological conditions. Thus, a contribution of systemic FSTL3 to breast cancer growth cannot be ruled out. In this context, the diagnostic and prognostic value of serum FSTL3 levels in breast cancer progression should be investigated in future studies.

We conclude that tumor tissue expression of FSTL3 is not a prognostic factor of clinical relevance for breast cancer patients. FSTL3 expression in invasive breast cancer is inversely associated with tumor size and nuclear tumor grade but it does not predict disease relapse in the short term.

## MATERIALS AND METHODS

### Patients and samples

This was a retrospective cohort study including 154 patients with breast cancer treated at Alberto Cavalcanti Hospital in Belo Horizonte, Brazil, between 2008 and 2012. The study was approved by the Ethics Committees of both the hospital and the university, and all participants provided written informed consent to have their surgical specimens banked and used in future research [24]. Patients who had been submitted to surgery (primary, n=132; or for recurrence, n=22), chemotherapy (adjuvant, neoadjuvant or palliative), hormonal therapy (adjuvant, neoadjuvant or palliative) and radiotherapy (adjuvant, neoadjuvant or palliative) were included in the study according to the guideline of the institution for invasive breast cancer treatment.

Clinical and pathological data were obtained from standard hospital records and clinical endpoints examined included age, menopausal status, disease stage, recurrence (local or systemic), histological type, tumor size, number of metastatic lymph nodes involved, histological grade (Bloom Richardson modified by Elston and Ellis), nuclear grade and mitotic index, estrogen receptor (ER), progesterone receptor (PR) and HER2 status, according to ASCO/CAP guideline [25]. Follistatin scores were available from a previous study of the same tumor specimens [24].

The clinical characteristics of the study participants are summarized in Table 4. Median age was 57 years (IQR 47-69 years), median tumor size was 18 mm (IQR 13-28 mm) and median number of metastatic lymph nodes was 1 (IQR 0-3 nodes), with 54% of the patients having at least one metastatic lymph node. Seventy-four percent of the participants were postmenopausal, 85% had ductal invasive breast cancer no otherwise specified (DIC NOS), 14% had skin invasion by the tumor, 32% were stage III or stage IV, and 13% had triple-negative receptor profile (Table 4).

**Table 4:** Clinical characteristics of the study participants

	Frequency	Median (IQR)
Age		57 (47-69)
Tumor size (mm)		18 (13-28)
Metastatic lymph nodes		1 (0-3)
Post-menopause	98/132 (74%)	
Histological type IDC NOS	130/153 (85%)	
Disease stage		
I	42/153 (27%)	
II	63/153 (41%)	
III	44/153 (29%)	
IV	4/153 (3%)	
Skin invasion	22/153 (14%)	
Receptor profile		
ER+, PR+, HER2+	28/119 (24%)	
ER+, PR+, HER2-	55/119 (46%)	
ER+, PR-, HER2-	10/119 (8%)	
ER-, PR-, HER2+	9/119 (8%)	
ER-, PR+, HER2-	1/119 (1%)	
ER-, PR-, HER2-	16/119 (13%)	

IDC NOS: ductal carcinoma not otherwise specified

### Immunohistochemistry assay for FSTL3 expression

Breast tumor samples previously fixed in buffered formalin and embedded in paraffin were cut into 5-μm sections that were mounted on gelatinized slides, deparaffinized and rehydrated through graded concentrations of ethanol, followed by endogenous peroxidase blockage by the use of 3% H_2_O_2_ in methanol solution. Then, for antigenic reactivation, sections were incubated for 5 minutes in pre-heated Tris buffer pH 9.0 and cooled at room temperature. Sections were washed in TBST buffer and incubated with normal rabbit serum for 1 h to block non-specific binding sites. The sections were then incubated with rabbit polyclonal primary antibody to human FSTL3 (LifeSpan Biosciences, Seattle, WA, USA, catalog # LS-C166265) at 1:60 dilution (final concentration 17 μg/mL, equivalent to 112 nMol/L) at room temperature for 1 hour, followed by HRP Polymer (Cell Marque, Roclin, CA, USA) and diaminobenzidine. This primary antibody was raised against a synthetic peptide corresponding to amino acids 229-258 from C-terminal region of human FSTL3. Tissue slides were counterstained with hematoxylin (Sigma Chemicals). The same breast cancer sample was used as positive control in all reactions and for negative control the slides were incubated with TBST buffer instead of primary antibody. Additional negative control was obtained by preadsorption of the primary antibody with equimolar concentration of a synthetic peptide specific to the human FSTL3 amino acid sequence (LifeSpan Biosciences, Seattle, WA, USA, catalog # LS-E1561.

Images were acquired and analyzed on a Carl Zeiss Axioplan 2 imaging microscope by AxioCam HR CCD camera and AxioVision 3.1 software (Carl Zeiss, Göttingen, Germany) by two different researchers blinded to patient identity. The intensity of nuclear staining in the tumoral epithelium (Figure 1) was graded on a 0-3 arbitrary unit scale as previously described [19, 26] and the percentage of tumoral cells with positive staining was graded 0 (absent), 1 (1% to 25%), 2 (26%-75%) or 3 (76%-100%). An index was obtained by summing the intensity and the percentage scores.

### Statistical analysis

Data are reported as medians and quartiles due to non-normal distribution of FSTL3 staining index. Two-group comparisons were carried out with Mann-Whitney test for continuous variables and Fisher's exact test for categorical variables, while linear correlations were tested with Spearman's rank correlation coefficients. Multivariate analysis was performed with backward stepwise logistic regression adopting a 95% statistical significance level.

Survival analysis was performed setting time to recurrence as time variable and FSTL3 staining and disease stage as factors; cases were censored when a recurrence was confirmed or the patient follow-up ended, whichever came first. The survival curves were built by the Kaplan-Meyer method and compared by the log-rank test, while the mean survival time was estimated with 95% confidence interval. The sample size was estimated to detect a 50% difference in the disease-free survival rate between patient groups at a two sided 0.05 significance level, with 90% statistical power [27].
